# TMPRSS2 and RNA-Dependent RNA Polymerase Are Effective Targets of Therapeutic Intervention for Treatment of COVID-19 Caused by SARS-CoV-2 Variants (B.1.1.7 and B.1.351)

**DOI:** 10.1128/spectrum.00472-21

**Published:** 2021-08-11

**Authors:** Jihye Lee, JinAh Lee, Hyeon Ju Kim, Meehyun Ko, Youngmee Jee, Seungtaek Kim

**Affiliations:** a Zoonotic Virus Laboratory, Institut Pasteur Korea, Seongnam, South Korea; b CEO office, Institut Pasteur Korea, Seongnam, South Korea; University of Nevada, Reno

**Keywords:** COVID-19, RNA-dependent RNA polymerase, SARS-CoV-2, TMPRSS2, variant

## Abstract

Severe acute respiratory syndrome coronavirus 2 (SARS-CoV-2) is a causative agent of the coronavirus disease 2019 (COVID-19) pandemic, and the development of therapeutic interventions is urgently needed. So far, monoclonal antibodies and drug repositioning are the main methods for drug development, and this effort was partially successful. Since the beginning of the COVID-19 pandemic, the emergence of SARS-CoV-2 variants has been reported in many parts of the world, and the main concern is whether the current vaccines and therapeutics are still effective against these variant viruses. Viral entry and viral RNA-dependent RNA polymerase (RdRp) are the main targets of current drug development; therefore, the inhibitory effects of transmembrane serine protease 2 (TMPRSS2) and RdRp inhibitors were compared among the early SARS-CoV-2 isolate (lineage A) and the two recent variants (lineage B.1.1.7 and lineage B.1.351) identified in the United Kingdom and South Africa, respectively. Our *in vitro* analysis of viral replication showed that the drugs targeting TMPRSS2 and RdRp are equally effective against the two variants of concern.

**IMPORTANCE** The COVID-19 pandemic is causing unprecedented global problems in both public health and human society. While some vaccines and monoclonal antibodies were successfully developed very quickly and are currently being used, numerous variants of the causative SARS-CoV-2 are emerging and threatening the efficacy of vaccines and monoclonal antibodies. In order to respond to this challenge, we assessed antiviral efficacy of small-molecule inhibitors that are being developed for treatment of COVID-19 and found that they are still very effective against the SARS-CoV-2 variants. Since most small-molecule inhibitors target viral or host factors other than the mutated sequence of the viral spike protein, they are expected to be potent control measures against the COVID-19 pandemic.

## INTRODUCTION

Coronavirus disease 2019 (COVID-19) is an emerging infectious disease caused by a novel coronavirus, severe acute respiratory syndrome coronavirus 2 (SARS-CoV-2) (1), and it was declared as a pandemic by the WHO on March 11, 2020. To address this unprecedented global challenge, intensive investigations have been simultaneously conducted by global scientific communities and industries to develop diagnostic tools, vaccines, and therapeutics. Remarkably, within 10 months after release of the SARS-CoV-2 genome sequence, a couple of vaccines were successfully developed and are now being used for vaccination of people after emergency use authorization (EUA). Drug development was also partially successful, especially in the development of monoclonal antibodies (2, 3). Notably, the vaccines and monoclonal antibodies currently being used are heavily dependent on the structure and sequence of viral spike protein, which is a surface glycoprotein responsible for virus entry and interacts with the host receptor angiotensin-converting enzyme 2 (ACE2). Thus, if there is any mutation in this protein, it is likely to affect the efficacy of both vaccines and antibodies.

**FIG 1:**
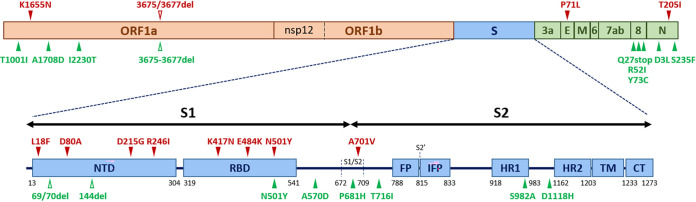
Schematic illustration of single-nucleotide polymorphisms (SNPs) in SARS-CoV-2 variants. Three SARS-CoV-2 lineages were used in this study: lineage A (an early SARS-CoV-2 isolate), lineage B.1.1.7 (identified in the United Kingdom), and lineage B.1.351 (identified in South Africa). SNPs that are observed in B.1.351 compared to the early isolate are noted in red above the diagram. SNPs observed in B.1.1.7 compared to the early isolate are noted in green below the diagram. NTD, N-terminal domain; RBD, receptor-binding domain; FP, fusion peptide; IFP, internal fusion peptide; HR1, heptad repeat 1; HR2, heptad repeat 2; TM, transmembrane anchor; CT, cytoplasmic tail; ORF, open reading frame; del, deletion.

Since the beginning of the COVID-19 pandemic, variants of SARS-CoV-2 have been reported in many parts of the world, and the recent variants identified in the United Kingdom (lineage B.1.1.7), South Africa (lineage B.1.351), and Brazil (lineage P.1) are of particular concern due to multiple mutations in the spike gene (Fig. 1) (4, 5). Indeed, several results are being published that demonstrated reduced neutralization capacity of convalescent plasma, vaccine sera, and monoclonal antibodies against these variants (6–9).

In addition to monoclonal antibodies, small-molecule inhibitors are also being developed as potential antiviral agents. Targets of such small-molecule inhibitors are often transmembrane serine protease 2 (TMPRSS2) (10–13) and viral RNA-dependent RNA polymerase (RdRp) (14, 15). TMPRSS2 is known to possess serine protease activity, which primes the viral spike protein for fusion between the viral membrane and the host cell membrane before the release of viral genome into the cytoplasm. Camostat and nafamostat are representative drug candidates as TMPRSS2 inhibitors and are currently being tested in several phase 2 and 3 clinical trials in many countries (NCT04623021, NCT04390594, NCT04483960, NCT04521296, NCT04721535, NCT04530617, etc.). On the other hand, RdRp is a target of remdesivir, which is the first approved drug for treatment of COVID-19 patients (16).

In this study, we investigated whether the antiviral drug candidates targeting TMPRSS2 and RdRp are still effective against the recent SARS-CoV-2 variants of concern by assessing *in vitro* viral replication capacity after drug treatment.

## RESULTS AND DISCUSSION

The alignment of SARS-CoV-2 amino acid sequences of two lineages (B.1.1.7 and B.1.351) identified numerous changes compared to the sequence of the early SARS-CoV-2 isolate (lineage A). Several changes were located in the spike protein (Fig. 1), while no change was observed in the NSP12 amino acid sequence, which possesses an RdRp activity.

**FIG 2:**
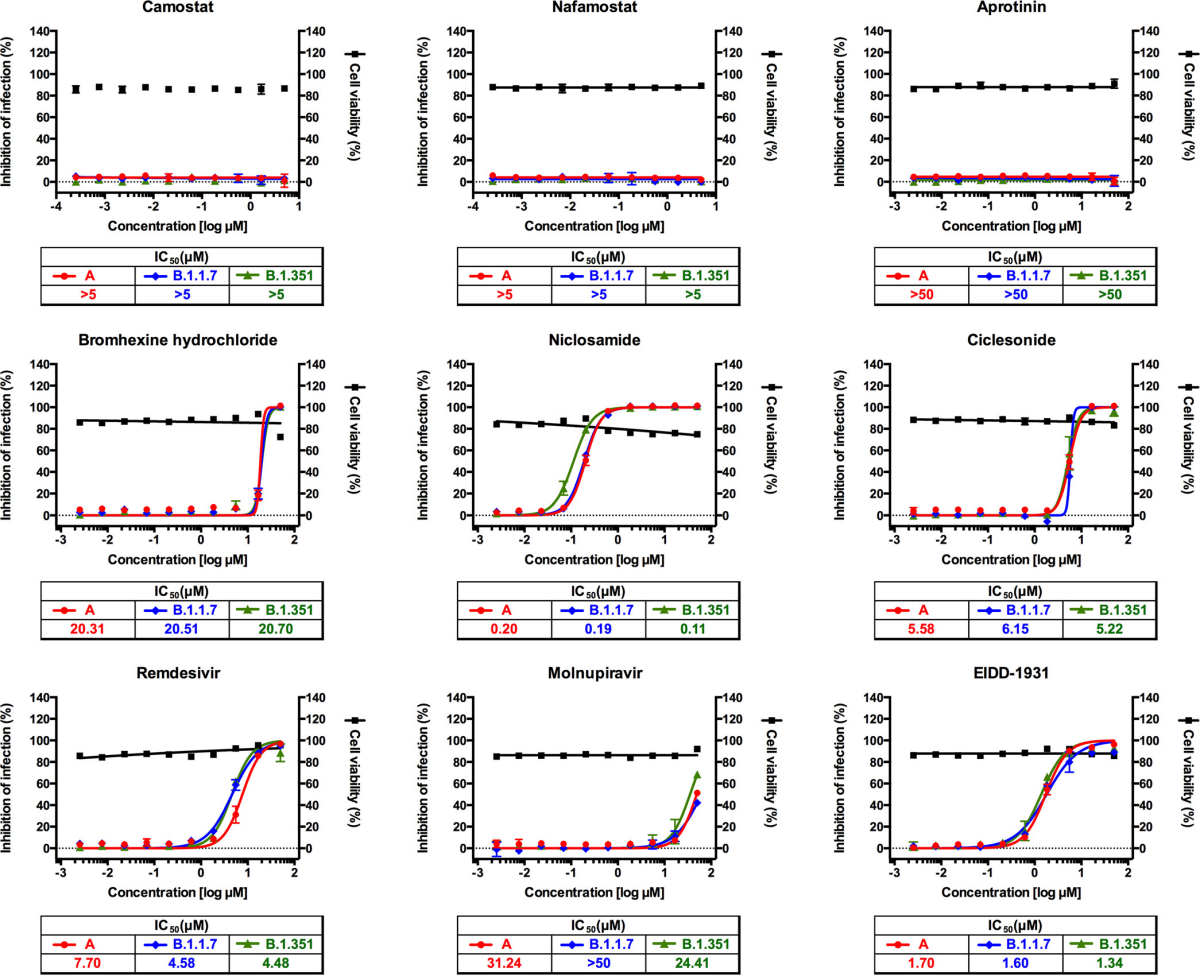
Dose-response curve analysis in Vero cells for the nine drugs that were tested in this study. The red circles (lineage A), blue diamonds (lineage B.1.1.7), and green triangles (lineage B.1.351) represent inhibition of SARS-CoV-2 infection (%) in the presence of increasing concentrations of each drug, and the black squares represent cell viability (%). In each panel, the symbols indicate actual data, while lines indicate the model fitting. Means ± SD were calculated from duplicate experiments.

**FIG 3:**
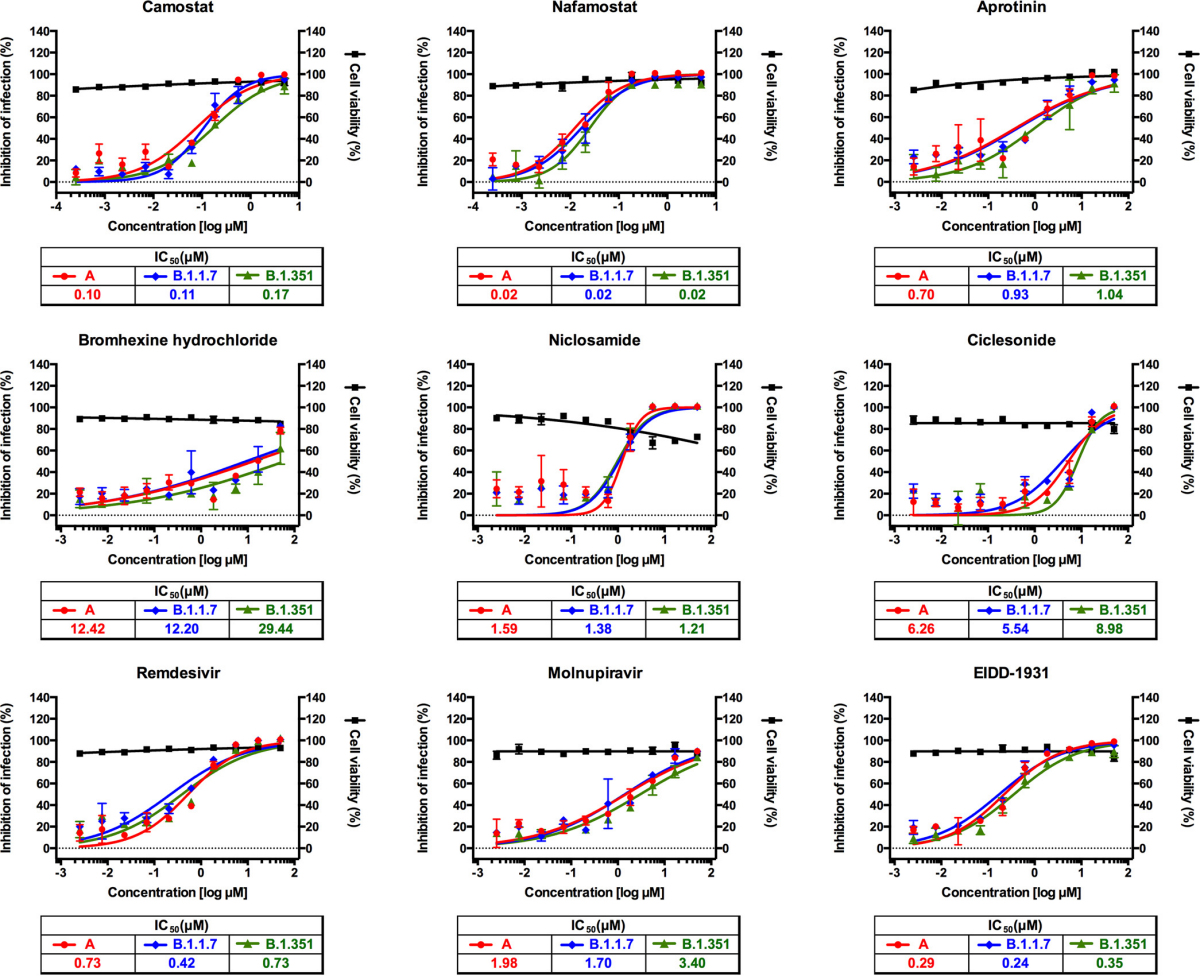
Dose-response curve analysis in Calu-3 cells for the nine drugs that were tested in this study. The red circles (lineage A), blue diamonds (lineage B.1.1.7), and green triangles (lineage B.1.351) represent inhibition of SARS-CoV-2 infection (%) in the presence of increasing concentrations of each drug, and the black squares represent cell viability (%). In each panel, the symbols indicate actual data, while lines indicate the model fitting. Means ± SD were calculated from duplicate experiments.

In order to compare drug efficacy against the three lineages of SARS-CoV-2, both Vero cells (Table 1) and Calu-3 cells (Table 2) were used for virus infection and drug treatment. Drugs were added to the cells before virus infection. Cells were fixed at 24 h postinfection and scored by immunofluorescence analysis with an antibody specific for the viral nucleocapsid (N) protein. For all drugs, cytotoxicity results were identical among the three variants, having a 50% cytotoxic concentration (CC_50_) value above the highest concentration tested (5 or 50 μM). The microscopic images of both viral N protein and cell nuclei were analyzed using Columbus software, and the dose-response curve (DRC) for each drug and variant was generated (Fig. 2 and 3).

**TABLE 1:** Comparison of IC_50_ values among three SARS-CoV-2 variants in Vero cells

Compound	A		B.1.1.7		B.1.351	
	Mean ± SD^*a*^ (μM)		Mean ± SD^*a*^ (μM)	*P* value^*b*^	Mean ± SD^*a*^ (μM)	*P* value^*b*^
Camostat	>5		>5	NA^*c*^	>5	NA
Nafamostat	>5		>5	NA	>5	NA
Aprotinin	>50		>50	NA	>50	NA
Bromhexine	20.31 ± 0.420		20.51 ± 0.017	0.526	20.70 ± 0.253	0.427
Niclosamide	0.204 ± 0.013		0.192 ± 0.007	0.348	0.114 ± 0.014	0.022
Ciclesonide	5.584 ± 0.348		6.154 ± 0.124	0.177	5.220 ± 0.315	0.961
Remdesivir	7.696 ± 0.646		4.583 ± 0.484	0.033	4.476 ± 0.107	0.021
Molnupiravir	31.24 ± 1.965		>50	NA	24.41 ± 4.140	0.219
EIDD-1931	1.699 ± 0.126		1.599 ± 0.116	0.557	1.336 ± 0.122	0.094

a Mean of 50% inhibitory concentration (IC_50_) values ± standard deviation (SD) of duplicate IC_50_ values.

b Student’s *t* test results by comparing IC_50_ values of A to IC_50_ values of B.1.1.7 or B.1.351.

c NA, not available.

**TABLE 2:** Comparison of IC_50_ values among three SARS-CoV-2 variants in Calu-3 cells

Compound	A		B.1.1.7		B.1.351	
	Mean ± SD^*a*^ (μM)		Mean ± SD^*a*^ (μM)	*P* value^*b*^	Mean ± SD^*a*^ (μM)	*P* value^*b*^
Camostat	0.102 ± 0.023		0.108 ± 0.006	0.797	0.170 ± 0.013	0.064
Nafamostat	0.016 ± 0.003		0.018 ± 0.009	0.650	0.024 ± 0.000	0.065
Aprotinin	0.696 ± 0.333		0.930 ± 0.108	0.457	1.037 ± 0.364	0.347
Bromhexine	12.42 ± 0.378		12.20 ± 0.392	0.836	29.44 ± 13.89	0.285
Niclosamide	1.587 ± 0.119		1.383 ± 0.179	0.475	1.210 ± 0.021	0.045
Ciclesonide	6.258 ± 1.384		5.541 ± 0.157	0.528	8.980 ± 2.235	0.462
Remdesivir	0.730 ± 0.061		0.423 ± 0.050	0.036	0.726 ± 0.052	0.652
Molnupiravir	1.979 ± 0. 161		1.705 ± 0.714	0.916	3.403 ± 0.030	0.007
EIDD-1931	0.292 ± 0.067		0.245 ± 0.014	0.476	0.351 ± 0.070	0.355

a Mean of 50% inhibitory concentration (IC_50_) values ± standard deviation (SD) of duplicate IC_50_ values.

b Student’s *t* test results by comparing IC_50_ values of A to IC_50_ values of B.1.1.7 or B.1.351.

We tested four different TMPRSS2 inhibitors (camostat, nafamostat, aprotinin, and bromhexine) (17), two RdRp inhibitors (remdesivir, EIDD-2801 [molnupiravir], and EIDD-1931 [an active form of EIDD-2801]) (14, 15), and others (niclosamide and ciclesonide) that we had identified in our earlier drug repositioning study (13, 18). The antiviral drug efficacy of each drug was compared among the three lineages of SARS-CoV-2, A (an early SARS-CoV-2 isolate), B.1.1.7 (identified in the United Kingdom), and B.1.351 (identified in South Africa).

While TMPRSS2 inhibitors did not show any antiviral effect in Vero cells as reported previously (Fig. 2) (13), they were very effective in suppressing viral replication in Calu-3 cells, perhaps due to the abundant TMPRSS2 expression in this cell line (19), without substantial differences in drug efficacy among the three lineages of SARS-CoV-2 (Fig. 3). TMPRSS2 cleaves the spike protein at the S2′ cleavage site, and no sequence change was observed at or near this site in the two recent variants (B.1.1.7 and B.1.351) compared to the sequence of the early SARS-CoV-2 isolate (lineage A) (Fig. 1). Perhaps, the conserved sequence at this region could account for the similar drug efficacy among the three lineages.

The amino acid sequence of NSP12 was also well conserved among the three lineages of SARS-CoV-2 (Fig. 1), and we did not find any substantial differences among them with regard to drug efficacy of the two representative RdRp inhibitors (remdesivir and molnupiravir) (Fig. 2 and 3). Both remdesivir and molnupiravir are nucleoside analogs; however, the two drugs differ from each other in that remdesivir works as a chain terminator but molnupiravir induces mutations during viral RNA replication. Molnupiravir (EIDD-2801) is a prodrug of β-d-*N*^4^-hydroxycytidine (EIDD-1931), and it has well-known broad-spectrum antiviral activity against various RNA viruses (20–23). Since this drug is orally available, it could be easily administered for patients even with mild COVID-19 if it is successfully developed. Currently, phase 2 and 3 clinical trials are being conducted globally for this new drug candidate (NCT04405739, NCT04575597, NCT04939428, etc.).

Finally, we assessed the antiviral drug efficacy of niclosamide and ciclesonide, and no substantial differences in drug efficacy were observed among the three lineages (Fig. 2 and 3). This result suggests that the potential targets of these drugs lie outside the substituted amino acids in the two variants. Currently, niclosamide and ciclesonide are being tested in several clinical trials to assess antiviral efficacy against SARS-CoV-2 infection (NCT04330586, NCT04749173, NCT04399356, etc.).

Most monoclonal antibodies, convalescent plasma, and vaccines that are being used for treatment or prevention of COVID-19 were developed to target the viral spike protein, specifically, the receptor-binding domain. While this protein is abundant and more immunogenic than the other viral proteins, it is also the place where many mutations occur (e.g., N501Y, E484K, and K417N) due to potential viral adaptations and various selective pressures, etc. Of these mutations, some are known to substantially reduce neutralization capacity of monoclonal antibodies, convalescent plasma, and vaccine sera. Hence, it is very important to develop therapeutics targeting viral or host factors other than the spike protein to address potential resistance issues caused by spike mutations.

In summary, we analyzed the efficacy of potential drug candidates (i.e., TMPRSS2 inhibitors, RdRp inhibitors, and others) against the recent SARS-CoV-2 variants of concern, and we found that all of them were equally effective in suppressing replication of B.1.1.7 and B.1.351 variants compared to the early SARS-CoV-2 isolate. The results from this study would help develop therapeutic interventions specifically targeting TMPRSS2, RdRp, or other viral and host factors.

## MATERIALS AND METHODS

### Virus and cells.

Vero and Vero E6 cells were obtained from ATCC (CCL-81 and C1008, respectively) and maintained at 37°C with 5% CO_2_ in Dulbecco’s modified Eagle’s medium (DMEM; Welgene) supplemented with 10% heat-inactivated fetal bovine serum (FBS) and 2% antibiotic-antimycotic solution (Gibco). The Calu‐3 cell line used in this study is a clonal isolate, which shows a higher growth rate than the parental Calu‐3 cell line obtained from ATCC (HTB‐55). Calu‐3 was maintained at 37°C with 5% CO_2_ in Eagle’s minimum essential medium (EMEM; ATCC) supplemented with 20% heat‐inactivated FBS, 1% MEM-nonessential amino acid solution (Gibco), and 2% antibiotic‐antimycotic solution (Gibco). Three lineages of SARS‐CoV‐2 were provided by the Korea Disease Control and Prevention Agency (KDCA) and were propagated in Vero E6 cells. Each lineage is noted as lineage A (an early SARS-CoV-2 isolate) (hCoV-19/Korea/KCDC03/2020), lineage B.1.1.7 (hCoV-19/Korea/KDCA51463/2021), and lineage B.1.351 (hCoV-19/Korea/KDCA55905/2021) in this study. Viral titers were determined by plaque assays in Vero cells (24). All experiments using SARS‐CoV‐2 were performed at Institut Pasteur Korea in compliance with the guidelines of the Korea National Institute of Health (KNIH) using enhanced biosafety level 3 (BSL‐3) containment procedures in laboratories approved for use by the KDCA.

### Reagents.

All compounds except for ciclesonide and EIDD-1931 were purchased from MedChemExpress (Monmouth Junction, NJ, USA). Ciclesonide and EIDD-1931 were purchased from Cayman Chemical (Ann Arbor, MI, USA). The following are the lot numbers and purities of each compound: aprotinin (lot, 62009; purity of ≥98.0%), bromhexine hydrochloride (lot, 15159; purity of 99.39%), niclosamide (lot, 15718; purity of 98.68%), ciclesonide (lot, 0472344-2; purity of ≥98.0%), remdesivir (lot, 46182; purity of 99.78%), EIDD-2801 (lot, 67548; purity of 99.94%), and EIDD-1931 (lot, 0590872-1; purity of ≥95.0%). Stock solution was dissolved in dimethyl sulfoxide (DMSO) at a 10 mM concentration. Anti‐SARS‐CoV‐2 N protein antibody was purchased from Sino Biological Inc. (Beijing, China). Alexa Fluor 488 goat anti‐rabbit IgG (H + L) secondary antibody and Hoechst 33342 were purchased from Molecular Probes. Paraformaldehyde (PFA) (32% aqueous solution) and normal goat serum were purchased from Electron Microscopy Sciences (Hatfield, PA, USA) and Vector Laboratories, Inc. (Burlingame, CA, USA), respectively.

### Dose-response curve analysis.

Vero cells were seeded at 1.0 × 10^4^ cells per well with DMEM (Welgene) supplemented with 2% heat-inactivated FBS and 2% antibiotic-antimycotic solution (Gibco) in black, 384‐well μClear plates (Greiner Bio‐One) 24 h before the experiment. Calu-3 cells were seeded at 2.0 × 10^4^ cells per well with EMEM (ATCC) supplemented with 20% heat-inactivated FBS, 1% MEM-nonessential amino acid solution (Gibco), and 2% antibiotic-antimycotic solution (Gibco) in black, 384‐well μClear plates (Greiner Bio‐One) 24 h before the experiment. The seeding density of Calu-3 cells was two times more than that for Vero cells due to the lower growth rate of Calu-3 cells. Ten‐point DRCs were generated with 3-fold dilutions, with compound concentrations ranging from 0.0025 to 50 μM. Only nafamostat and camostat used a top concentration of 5 μM instead of 50 μM; thus, concentrations ranged from 0.00025 to 5 μM. For viral infection, plates were transferred into the BSL‐3 containment facility, and SARS‐CoV‐2 was added at a multiplicity of infection of 0.008 for Vero cells and 0.2 for Calu-3 cells. The plates were incubated at 37°C for 24 h. The cells were fixed at 24 h postinfection with 4% PFA and permeabilized with 0.25% Triton X-100 solution. Anti-SARS-CoV-2 nucleocapsid (N) primary antibody, 488-conjugated goat anti-rabbit IgG secondary antibody, and Hoechst 33342 were applied to the cells for immunofluorescence. Images acquired with an Operetta high-throughput imaging device (PerkinElmer) were analyzed using Columbus software (PerkinElmer) to quantify cell numbers and infection ratios. Antiviral activity was normalized to infection control (0.5% DMSO) in each assay plate. Cell viability was measured by counting nuclei in each well and normalizing it to the mock control. The plots for DRCs were generated using Prism7 software (GraphPad, San Diego, CA, USA). The 50% inhibitory concentration (IC_50_) and 50% cytotoxic concentration (CC_50_) values were calculated using nonlinear regression analysis, log(inhibitor concentration) versus response − variable slope (four parameters) with the following equation: *Y* = bottom + (top − bottom)/(1 + 10^[(logIC50 –^ *^X^*^) × HillSlope]^). All IC_50_ and CC_50_ values were measured in duplicate.

## References

[1] Zhou P, Yang X-L, Wang X-G, Hu B, Zhang L, Zhang W, Si H-R, Zhu Y, Li B, Huang C-L, Chen H-D, Chen J, Luo Y, Guo H, Jiang R-D, Liu M-Q, Chen Y, Shen X-R, Wang X, Zheng X-S, Zhao K, Chen Q-J, Deng F, Liu L-L, Yan B, Zhan F-X, Wang Y-Y, Xiao G-F, Shi Z-L. 2020. A pneumonia outbreak associated with a new coronavirus of probable bat origin. Nature 579:270–273. doi:10.1038/s41586-020-2012-7. 32015507 PMC7095418

[2] Chen P, Nirula A, Heller B, Gottlieb RL, Boscia J, Morris J, Huhn G, Cardona J, Mocherla B, Stosor V, Shawa I, Adams AC, Van Naarden J, Custer KL, Shen L, Durante M, Oakley G, Schade AE, Sabo J, Patel DR, Klekotka P, Skovronsky DM, BLAZE-1 Investigators. 2021. SARS-CoV-2 neutralizing antibody LY-CoV555 in outpatients with Covid-19. N Engl J Med 384:229–237. doi:10.1056/NEJMoa2029849. 33113295 PMC7646625

[3] Weinreich DM, Sivapalasingam S, Norton T, Ali S, Gao H, Bhore R, Musser BJ, Soo Y, Rofail D, Im J, Perry C, Pan C, Hosain R, Mahmood A, Davis JD, Turner KC, Hooper AT, Hamilton JD, Baum A, Kyratsous CA, Kim Y, Cook A, Kampman W, Kohli A, Sachdeva Y, Graber X, Kowal B, DiCioccio T, Stahl N, Lipsich L, Braunstein N, Herman G, Yancopoulos GD, Trial Investigators. 2021. REGN-COV2, a neutralizing antibody cocktail, in outpatients with Covid-19. N Engl J Med 384:238–251. doi:10.1056/NEJMoa2035002. 33332778 PMC7781102

[4] Fontanet A, Autran B, Lina B, Kieny MP, Karim SSA, Sridhar D. 2021. SARS-CoV-2 variants and ending the COVID-19 pandemic. Lancet 397:952–954. doi:10.1016/S0140-6736(21)00370-6. 33581803 PMC7906631

[5] Mascola JR, Graham BS, Fauci AS. 2021. SARS-CoV-2 viral variants—tackling a moving target. JAMA 325:1261–1262. doi:10.1001/jama.2021.2088. 33571363

[6] Greaney AJ, Loes AN, Crawford KHD, Starr TN, Malone KD, Chu HY, Bloom JD. 2021. Comprehensive mapping of mutations in the SARS-CoV-2 receptor-binding domain that affect recognition by polyclonal human plasma antibodies. Cell Host Microbe 29:463–476. doi:10.1016/j.chom.2021.02.003. 33592168 PMC7869748

[7] Liu Z, VanBlargan LA, Bloyet L-M, Rothlauf PW, Chen RE, Stumpf S, Zhao H, Errico JM, Theel ES, Liebeskind MJ, Alford B, Buchser WJ, Ellebedy AH, Fremont DH, Diamond MS, Whelan SPJ. 2021. Identification of SARS-CoV-2 spike mutations that attenuate monoclonal and serum antibody neutralization. Cell Host Microbe 29:477–488. doi:10.1016/j.chom.2021.01.014. 33535027 PMC7839837

[8] Thomson EC, Rosen LE, Shepherd JG, Spreafico R, da Silva Filipe A, Wojcechowskyj JA, Davis C, Piccoli L, Pascall DJ, Dillen J, Lytras S, Czudnochowski N, Shah R, Meury M, Jesudason N, De Marco A, Li K, Bassi J, O’Toole A, Pinto D, Colquhoun RM, Culap K, Jackson B, Zatta F, Rambaut A, Jaconi S, Sreenu VB, Nix J, Zhang I, Jarrett RF, Glass WG, Beltramello M, Nomikou K, Pizzuto M, Tong L, Cameroni E, Croll TI, Johnson N, Di Iulio J, Wickenhagen A, Ceschi A, Harbison AM, Mair D, Ferrari P, Smollett K, Sallusto F, Carmichael S, Garzoni C, Nichols J, Galli M, et al. 2021. Circulating SARS-CoV-2 spike N439K variants maintain fitness while evading antibody-mediated immunity. Cell 184:1171–1187. doi:10.1016/j.cell.2021.01.037. 33621484 PMC7843029

[9] Wang Z, Schmidt F, Weisblum Y, Muecksch F, Barnes CO, Finkin S, Schaefer-Babajew D, Cipolla M, Gaebler C, Lieberman JA, Oliveira TY, Yang Z, Abernathy ME, Huey-Tubman KE, Hurley A, Turroja M, West KA, Gordon K, Millard KG, Ramos V, Da Silva J, Xu J, Colbert RA, Patel R, Dizon J, Unson-O’Brien C, Shimeliovich I, Gazumyan A, Caskey M, Bjorkman PJ, Casellas R, Hatziioannou T, Bieniasz PD, Nussenzweig MC. 2021. mRNA vaccine-elicited antibodies to SARS-CoV-2 and circulating variants. Nature 592:616–617. doi:10.1038/s41586-021-03324-6. 33567448 PMC8503938

[10] Hoffmann M, Kleine-Weber H, Schroeder S, Krüger N, Herrler T, Erichsen S, Schiergens TS, Herrler G, Wu N-H, Nitsche A, Müller MA, Drosten C, Pöhlmann S. 2020. SARS-CoV-2 cell entry depends on ACE2 and TMPRSS2 and is blocked by a clinically proven protease inhibitor. Cell 181:271–280. doi:10.1016/j.cell.2020.02.052. 32142651 PMC7102627

[11] Hoffmann M, Schroeder S, Kleine-Weber H, Müller MA, Drosten C, Pöhlmann S. 2020. Nafamostat mesylate blocks activation of SARS-CoV-2: new treatment option for COVID-19. Antimicrob Agents Chemother 64:e00754-20. doi:10.1128/AAC.00754-20. 32312781 PMC7269515

[12] Yamamoto M, Kiso M, Sakai-Tagawa Y, Iwatsuki-Horimoto K, Imai M, Takeda M, Kinoshita N, Ohmagari N, Gohda J, Semba K, Matsuda Z, Kawaguchi Y, Kawaoka Y, Inoue J. 2020. The anticoagulant nafamostat potently inhibits SARS-CoV-2 S protein-mediated fusion in a cell fusion assay system and viral infection *in vitro* in a cell-type-dependent manner. Viruses 12:629. doi:10.3390/v12060629. 32532094 PMC7354595

[13] Ko M, Jeon S, Ryu W, Kim S. 2021. Comparative analysis of antiviral efficacy of FDA‐approved drugs against SARS‐CoV‐2 in human lung cells. J Med Virol 93:1403–1408. doi:10.1002/jmv.26397. 32767684 PMC7436731

[14] Sheahan TP, Sims AC, Graham RL, Menachery VD, Gralinski LE, Case JB, Leist SR, Pyrc K, Feng JY, Trantcheva I, Bannister R, Park Y, Babusis D, Clarke MO, Mackman RL, Spahn JE, Palmiotti CA, Siegel D, Ray AS, Cihlar T, Jordan R, Denison MR, Baric RS. 2017. Broad-spectrum antiviral GS-5734 inhibits both epidemic and zoonotic coronaviruses. Sci Transl Med 9:eaal3653. doi:10.1126/scitranslmed.aal3653. 28659436 PMC5567817

[15] Sheahan TP, Sims AC, Zhou S, Graham RL, Pruijssers AJ, Agostini ML, Leist SR, Schäfer A, Dinnon KH, Stevens LJ, Chappell JD, Lu X, Hughes TM, George AS, Hill CS, Montgomery SA, Brown AJ, Bluemling GR, Natchus MG, Saindane M, Kolykhalov AA, Painter G, Harcourt J, Tamin A, Thornburg NJ, Swanstrom R, Denison MR, Baric RS. 2020. An orally bioavailable broad-spectrum antiviral inhibits SARS-CoV-2 in human airway epithelial cell cultures and multiple coronaviruses in mice. Sci Transl Med 12:eabb5883. doi:10.1126/scitranslmed.abb5883. 32253226 PMC7164393

[16] Beigel JH, Tomashek KM, Dodd LE, Mehta AK, Zingman BS, Kalil AC, Hohmann E, Chu HY, Luetkemeyer A, Kline S, Lopez de Castilla D, Finberg RW, Dierberg K, Tapson V, Hsieh L, Patterson TF, Paredes R, Sweeney DA, Short WR, Touloumi G, Lye DC, Ohmagari N, Oh M, Ruiz-Palacios GM, Benfield T, Fätkenheuer G, Kortepeter MG, Atmar RL, Creech CB, Lundgren J, Babiker AG, Pett S, Neaton JD, Burgess TH, Bonnett T, Green M, Makowski M, Osinusi A, Nayak S, Lane HC, ACTT-1 Study Group Members. 2020. Remdesivir for the treatment of Covid-19—final report. N Engl J Med 383:1813–1826. doi:10.1056/NEJMoa2007764. 32445440 PMC7262788

[17] Shen LW, Mao HJ, Wu YL, Tanaka Y, Zhang W. 2017. TMPRSS2: a potential target for treatment of influenza virus and coronavirus infections. Biochimie 142:1–10. doi:10.1016/j.biochi.2017.07.016. 28778717 PMC7116903

[18] Jeon S, Ko M, Lee J, Choi I, Byun SY, Park S, Shum D, Kim S. 2020. Identification of antiviral drug candidates against SARS-CoV-2 from FDA-approved drugs. Antimicrob Agents Chemother 64:e00819-20. doi:10.1128/AAC.00819-20. 32366720 PMC7318052

[19] Murgolo N, Therien AG, Howell B, Klein D, Koeplinger K, Lieberman LA, Adam GC, Flynn J, McKenna P, Swaminathan G, Hazuda DJ, Olsen DB. 2021. SARS-CoV-2 tropism, entry, replication, and propagation: considerations for drug discovery and development. PLoS Pathog 17:e1009225. doi:10.1371/journal.ppat.1009225. 33596266 PMC7888651

[20] Reynard O, Nguyen X-N, Alazard-Dany N, Barateau V, Cimarelli A, Volchkov V. 2015. Identification of a new ribonucleoside inhibitor of Ebola virus replication. Viruses 7:6233–6240. doi:10.3390/v7122934. 26633464 PMC4690858

[21] Urakova N, Kuznetsova V, Crossman DK, Sokratian A, Guthrie DB, Kolykhalov AA, Lockwood MA, Natchus MG, Crowley MR, Painter GR, Frolova EI, Frolov I. 2018. β-d-*N*^4^-Hydroxycytidine is a potent anti-alphavirus compound that induces a high level of mutations in the viral genome. J Virol 92:e01965-17. doi:10.1128/JVI.01965-17. 29167335 PMC5774879

[22] Toots M, Yoon JJ, Cox RM, Hart M, Sticher ZM, Makhsous N, Plesker R, Barrena AH, Reddy PG, Mitchell DG, Shean RC, Bluemling GR, Kolykhalov AA, Greninger AL, Natchus MG, Painter GR, Plemper RK. 2019. Characterization of orally efficacious influenza drug with high resistance barrier in ferrets and human airway epithelia. Sci Transl Med 11:eaax5866. doi:10.1126/scitranslmed.aax5866. 31645453 PMC6848974

[23] Agostini ML, Pruijssers AJ, Chappell JD, Gribble J, Lu X, Andres EL, Bluemling GR, Lockwood MA, Sheahan TP, Sims AC, Natchus MG, Saindane M, Kolykhalov AA, Painter GR, Baric RS, Denison MR. 2019. Small-molecule antiviral β-d-*N*^4^-hydroxycytidine inhibits a proofreading-intact coronavirus with a high genetic barrier to resistance. J Virol 93:e01348-19. doi:10.1128/JVI.01348-19. 31578288 PMC6880162

[24] Mendoza EJ, Manguiat K, Wood H, Drebot M. 2020. Two detailed plaque assay protocols for the quantification of infectious SARS-CoV-2. Curr Protoc Microbiol 57:ecpmc105. doi:10.1002/cpmc.105. 32475066 PMC7300432

